# Measurement Method for Contact Wire Wear Based on Stereovision

**DOI:** 10.3390/s24072085

**Published:** 2024-03-25

**Authors:** Wei Zhou, Zhe Qin, Xinyu Du, Xiantang Xue, Haiying Wang, Hailang Li

**Affiliations:** Infrastructure Inspection Research Institute, China Academy of Railway Sciences Corporation Limited, Beijing 100081, China

**Keywords:** contact wire wear, residual thickness, optical measurement, stereovision

## Abstract

The contact wire wear is an important parameter to ensure the safety operation of electric railways. The contact wire may break if the wear is serious, which leads to transportation interruptions. This study proposes an optical measurement method of contact wire wear, using stereovision technology. The matching method of stereovision based on line-scan cameras is proposed. A lookup-table method is developed to exactly determine the image resolution caused by the contact wire being in different spatial positions. The wear width of the contact wire is extracted from catenaries’ images, and the residual thickness of the contact wire is calculated. The method was verified by field tests. The round-robin tests of the residual thickness at the same location present excellent measurement repetitiveness. The maximum difference value between dynamic test results and ground measurement results is 0.13 mm. This research represents a potential way to implement condition-based maintenance for contact wire wear in the future in order to improve the maintenance efficiency and ensure the safety of catenary infrastructure.

## 1. Introduction

The contact wire is one of the most important components in an electrified railway system. It makes direct contact with the pantograph of the electric locomotive and supplies current to the locomotive. Because of the continuous friction between the pantograph and the contact wire, the wire surface wears out, thus reducing the effective cross-section of the wire [1,2,3]. The reduction of the cross-section causes different problems. Firstly, the mechanical strength of the contact wire reduces. If the cross-section is too small, the contact wire may break, which might cause hazardous accidents. Secondly, the resistance increases as the cross-section becomes smaller, and the voltage reduces which may not be able to meet the operational needs of electric locomotives. As a result, the contact wire wear should be monitored regularly to ensure the safety of the catenary infrastructure. If a section of contact wire with serious wear is detected at an early stage, this section of contact wire could be strengthened or replaced by a new section. Therefore, the lifetime of contact wire could be extended. In the past few decades, the measurement of contact wire wear has mainly relied on the method of manual measurement. For the manual measurement method, the safety risk for the maintenance workers is high; the power on the catenary has to be cut off, which leads to the traffic interruption; and the inspection workload is heavy because of the point-by-point measurement way. With the development of the high-speed electrified railway, the requirements were put forward to develop the dynamic inspection method of contact wire wear. The automatic measurement method of contact wire wear on rolling stock improves the maintenance efficiency and reduces the maintenance cost, because of the timely and fast defect detection. Furthermore, the continuous wear measurement along the railway line helps the railway operators to diagnose the abnormal function state, evaluate the quality of catenary, and implement the condition-based maintenance of catenary.

The contact wire height, stagger, and wear are the primary geometrical properties of the contact wire. The height and stagger describe the spatial position of the contact wire relative to the rail surface. The residual thickness indicates the degree of contact wire wear. With the rapid development of sensor technology in the past few decades, various measurement methods for the geometry parameters of contact wire have been developed [4,5,6,7], such as the microwave reflection analysis and electrical and optical methods. Among them, the optical method has been the most successful one to perform the measurement of contact wire wear, which includes the residual thickness measurement method, the laser scanning method, and vision measurement method [6]. The residual thickness measurement method presents a few limitations, such as the necessity of modifying the pantograph, a limited measurement range, and the fact that the maximum number of detectable wires is only one [6,7]. The obstacles of the laser scanning method are the limitation of the total measurement points per second, and it is sensitive to the reflectivity of contact wires. For the vision measurement method, the wear surface of contact wire is illuminated, and the wear width is extracted by analyzing the image taken by the cameras. Several vision measurement methods were reported, such as structured light vision measurement [8,9,10] and stereovision measurement [11,12,13]. For example, researchers from RTRI developed a contact wire wear measurement system based on structured light vision measurement [8]. The RTRI’s system was installed on a Shinkansen vehicle and was capable of an accuracy of within ±0.1 mm in residual thickness. Chugui et al. [9] developed a structured light vision measurement system with an RMS error of the residual thickness of 0.15 mm and a measurement rate of ~150 Hz. Nie [10] adopted a 2D laser sensor (ELAG Elektronik AG, Winterthur, Switzerland) to measure the wire wear of metro rigid catenary.

The limitations of structured light vision measurement are the low measurement frequency and the influence of sunlight in daytime inspection.

Stereovision measurement using two or more line-scan cameras is a better solution for the measurement of contact wire wear on rolling stock [7,14]. The advantages of stereovision measurement are the high scanning frequency and the high resolution because of the one-dimensional imaging chip of the line-scan camera. For this application, the scanning plane of the line-scan camera is perpendicular to the direction of the train. However, it is still a challenging work since it has to overcome several obstacles, such as the image quality during day and night, the measurement resolution, and the variations in both contact wire height and stagger [7,14]. Great efforts have been made to overcome the above challenges. Laser diodes combined with interference filters are used as the active light source, which improves the image SNR (signal-noise ratio) in daytime inspection [7,12]. Due to considerations of operational safety and laser temperature control, the power of the laser needs to be carefully designed. In order to cover the range of stagger with high resolution, multiple line-scan cameras were adopted by ATON and MEDES [7]. To minimize the influence of the variation in contact wire height on measurement, Van Gigch et al. [15] and Borromeo et al. [7] proposed a method of adjusting the focal lengths of cameras online separately, while Kusumi et al. [16] developed a method for keeping a constant distance from cameras to contact wire by controlling the mirrors. You et al. [12] derived the equation of calculating the wear width using the pinhole camera model, which involved the impact of height variation on resolution. On the other hand, stereo matching is another challenge in stereovision measurement. For a binocular vision system, stereo matching can be defined as finding the corresponding points between the stereo image pairs. Interest point detectors and feature descriptors are commonly used methods for solving the stereo-matching problem [14]. Zhou et al. [17] proposed a trinocular stereovision system that includes a projector and two area-scan cameras and utilized the phase information of pixels to complete the matching of homonymous points. Shu et al. [18] designed a trinocular stereovision system by using three area-scan cameras arranged in equilateral triangles and solved the matching problem by using the method of epipolar constraint with three intersecting cameras.

The author previously studied the method based on multi-view stereovision to measure the height and stagger of contact wire [19,20]. Four CCD line-scan cameras with a resolution of 4096 pixels and different angles and four high power spotlights (575 W for each spotlight) were adopted to form a robust system under most of the environmental conditions. If one of the four cameras was influenced by the direct sunlight, the other two cameras were used to perform the measurement. However, since the spectrum of the spotlight is wide and continuous, the strong sunlight is hard to weaken, and the grayscale feature of the wear surface could be hardly highlighted because of the poor image SNR. The contact wire in the image is usually a black object against a bright background in daytime inspection, thus making it difficult to measure the contact wire wear. Based on previous research, this paper proposes a contactless optical measurement method of contact wire wear, using the multi-view stereovision technology. A matching method of stereovision based on line-scan cameras is proposed. The third line-scan camera is adopted to overcome the correspondence problem. Compared with the matching method of binocular stereovision in the literature, this matching method is fast and robust, which satisfies the requirement of real-time measurement in day and night inspection. The wear width of the contact wire is extracted from catenaries’ images. A lookup-table method is developed to exactly determine the image resolution in various sections of measurement plane. Compared with the methods reported in the literature [7,15,16], no additional adjustment device is required to adjust the focal lengths of cameras or maintain a constant working distance in this study. Currently, little attention is being paid to the impact of stagger variation on resolution. In most existing studies, the focus is on minimizing the influence of the variation in contact wire height on measurement [7,15,16] or on evaluating the impact of height variation on resolution [12]. In this study, the impact of both the height variation and the stagger variation on the image resolution has been taken into account by using the lookup-table method, which ensures the accuracy of the wear measurement of contact wires. Then, the residual thickness is calculated according to the rated cross-sectional parameters of contact wire. In order to highlight the grayscale feature of the wear surface and improve the image SNR in daytime inspection, a new kind of high-speed synchronized stroboscopic lighting technology using monochromatic LED lamps combined with narrow band-pass optical filters is developed as an alternative active lighting method. Compared with the laser diode lighting method reported in the literature [7,12], the stroboscopic LED lighting method avoids the risk of laser operation and is harmless to nearby people. In addition, there is no need to design a precise mechanical adjustment device to make the scanning plane of the line-scan camera coincide with the laser irradiation plane.

The remaining sections of this paper are organized as follows: Section 2 introduces the basic principle of contact wire wear measurement using the vision measurement method. The measurement model and implementation method are detailed in Section 3. The test results and a discussion are given in Section 4. Finally, the work of the full paper is summarized, and conclusions and suggestions are given.

## 2. Basic Principle

**Figure 1:**
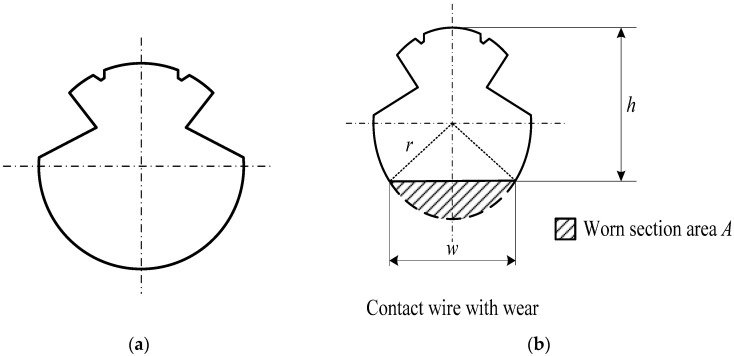
Cross-section of a contact wire: (**a**) a new contact wire and (**b**) a contact wire with wear.

Figure 1 shows the cross-section of a new contact wire (left side) and the cross-section of a contact wire with wear (right side). The key feature to be measured may be classified as the residual thickness (*h*) or the width of the wear surface (*w*), as can be seen in Figure 1b. For most railway companies, the residual thickness (*h*) or the worn section area (*A*) of the contact wire is used as the indicator of the degree of wire wear.

As shown in Figure 1, the lower half part of the cross-section of the wire is arc-shaped. In the case of a circular contact wire with radius, *r*, the residual thickness (*h*) can be calculated from the width of the wear surface, *w*:

(1) $$h=r+\sqrt{r^{2}-{\left(\frac{w}{2}\right)}^{2}}$$

The relationship between the worn section area, *A*, and the width of the wear surface, *w*, can be described by [7,21]:

(2) $${A=r}^{2}\sin^{-1}(\frac{w}{2r})-\frac{w}{2}\sqrt{r^{2}-{\left(\frac{w}{2}\right)}^{2}}$$

**Figure 2:**
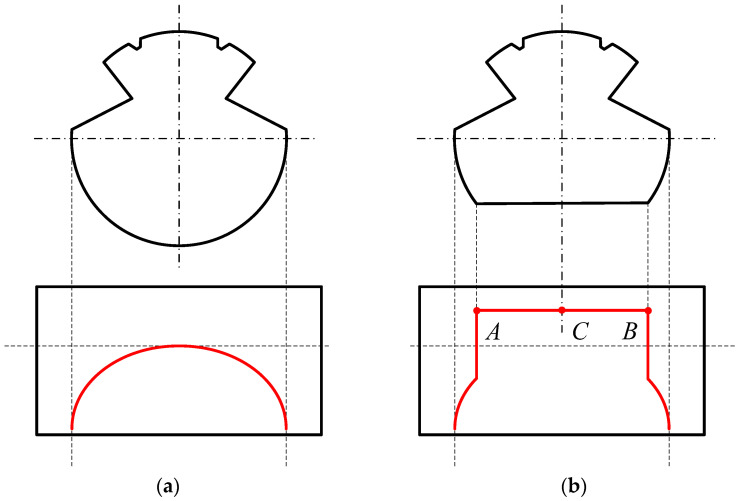
Grayscale curves (red lines) of cross-section of contact wires: (**a**) a new contact wire and (**b**) a contact wire with wear.

The contact wire wear measurement using the vision measurement method is based on the fact that the wearing surface of the contact wire presents good light-reflecting characteristics. The wearing surface is illuminated by the active lighting source, i.e., spotlights, laser diodes, and LED. Because of the continuous friction between the pantograph and the contact wire, the wearing surface is flat and has much greater light-reflecting characteristics than the lateral surface of the contact wire. In common, the grayscale of the wear surface region is much higher than the rest of the region of the contact wire. The red lines in Figure 2 show the grayscale curve of a new contact wire (Figure 2a) and the grayscale curve of a contact wire with wear (Figure 2b). Appropriate image-processing techniques are adopted to extract the key points, including the edge points and the central point of the wear surface, such as the Sobel operator and cross-correlation template matching. The width of the wear surface is the distance between the two edge points (points *A* and *B* in Figure 2b), indicating the residual geometric thickness of the contact wire. The central point of the wear surface (point *C* in Figure 2b) is usually chosen to calculate the values of height and stagger.

## 3. Materials and Methods

### 3.1. Stereovision Measurement Model

**Figure 3:**
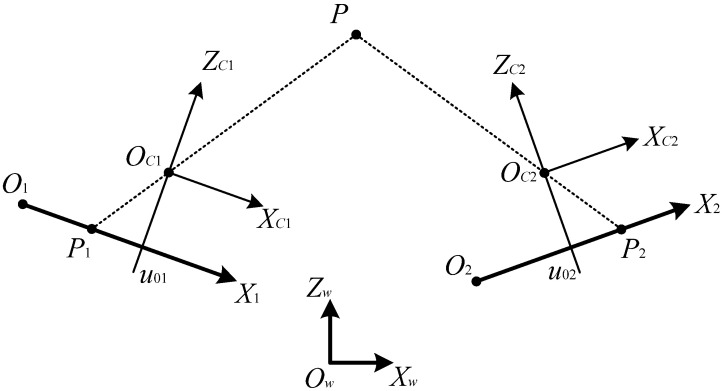
Measurement principle of the contact wire geometry parameters using stereovision method with line-scan cameras.

Since the image resolution varies with both the height and stagger values of the contact wire, the measurement of height and stagger is the basis of the measurement of contact wire wear. The measurement principle of the contact wire geometry parameters using the stereovision method is shown in Figure 3. In the figure, *O*_w_*X*_w_*Z*_w_ is the world coordinate system. The origin of this coordinate system, *O*_w_, is set at the midpoint of the train roof, and *Y*_w_ represents the direction of the train. *O*_1_*X*_1_ and *O*_2_*X*_2_ are the imaging coordinate systems of the left and right cameras, respectively. *O*_c1_*X*_c1_*Z*_c1_ and *O*_c2_*X*_c2_*Z*_c2_ are the camera coordinate systems of the left and right cameras, respectively. For these two camera coordinate systems, each origin is located at the principal point of the camera lens. The point *P* is projected onto the imaging chip of the line-scan camera through the optical lens. The imaging locations of the point *P* on the two cameras are *P*_1_ and *P*_2_, respectively. *U*_01_ and *u*_02_ are the image coordinates of the principal points of the two camera lenses.

By using perspective projection transformation, the transformation relation between the camera imaging coordinate system and the camera coordinate system is shown in Equation (3):

(3) $$s\left[\begin{array}{c} u\\ 1 \end{array}\right]=M_{1}\left[\begin{array}{c} X_{c}\\ Z_{c} \end{array}\right]$$

where *s* is the non-zero scale factor; u is the coordinate of image point of point *P* in any camera imaging coordinate system; [*X*_c_, *Z*_c_] is the coordinate of point *P* in the corresponding camera coordinate system; and ***M***_1_ is the internal parameter matrix of the camera and is given by the following:

(4) $$M_{1}=\left[\begin{array}{ccc} f_{e} & u_{0} & 0\\ 0 & 1 & 0 \end{array}\right]$$

In Equation (4), *f*_e_ is the normalized focal length of the camera lens, and *u*_0_ is the image coordinate of the principal point of the camera lens.

By using Euclid-space transformation, the transformation relation between the camera coordinate system and the world coordinate system is as shown below [22]:

(5) $$\left[\begin{array}{c} X_{c}\\ Z_{c} \end{array}\right]=M_{2}\left[\begin{array}{c} X_{w}\\ Z_{w} \end{array}\right]=\left[\begin{array}{cc} R & T\\ 0 & 1 \end{array}\right]\left[\begin{array}{c} X_{w}\\ Z_{w} \end{array}\right]$$

where [*X*_w_, *Z*_w_] is the coordinate of point *P* in the world coordinate system; ***M***_2_ is the external parameter matrix of the camera; and ***R*** and ***T*** are the rotation matrix and translation vector, respectively.

Combining Equation (3) with (5), the line-scan camera model is given by the following:

(6) $$s\left[\begin{array}{c} u\\ 1 \end{array}\right]=M_{1}\left[\begin{array}{c} X_{c}\\ Z_{c} \end{array}\right]=M_{1}M_{2}\left[\begin{array}{c} X_{w}\\ Z_{w} \end{array}\right]=M\left[\begin{array}{c} X_{w}\\ Z_{w} \end{array}\right]$$

where ***M*** is the intrinsic parameter matrix. ***M*** is described by the following:

(7) $$M=M_{1}M_{2}=\left[\begin{array}{ccc} m_{11} & m_{12} & m_{13}\\ m_{21} & m_{22} & m_{23} \end{array}\right]$$

By eliminating the variables in Equation (6), we can obtain the equation as follows:

(8) $$u=\frac{m_{11}X_{w}+m_{12}Z_{w}+m_{13}}{m_{21}X_{w}+m_{22}Z_{w}+m_{23}}$$

Equation (8) is the perspective projection equation of the line-scan camera, which describes the relationship between the coordinate of point *P* in the world coordinate system and the image coordinate of point *P* in the camera imaging coordinate system.

Multiple sets of calibration point data are obtained within the measurement range, including the world coordinate data, [*X*_w_, *Z*_w_], and the corresponding imaging coordinate data, *u*. Then, the parameters of the matrix, ***M***, in Equation (8) are calculated using mathematical procedures.

In principle, the coordinate of point *P* in the world coordinate system, [*X*_w_, *Z*_w_], could be calculated by using the imaging coordinate data, *u*, of any two line-scan cameras. The subscripts a and b are used to represent the left and right cameras in Figure 3, respectively. By combining the perspective projection equations of the two cameras, *X*_w_ and *Z*_w_ are given by the following:

(9) $$X_{w}=\frac{q_{2}k_{1}-q_{1}k_{2}}{p_{1}q_{2}-p_{2}q_{1}}\;Z_{w}=\frac{p_{1}k_{2}-p_{2}k_{1}}{p_{1}q_{2}-p_{2}q_{1}}$$

The parameters *p*_1_, *p*_2_, *q*_1_, *q*_2_, *k*_1_, and *k*_2_ in Equation (9) are given by the following:

(10) $$p_{1}=m_{11a}-u_{a}m_{21a}\;p_{2}=m_{11b}-u_{b}m_{21b}\;q_{1}=m_{12a}-u_{a}m_{22a}\;q_{2}=m_{12b}-u_{b}m_{22b}\;k_{1}={u_{a}m}_{23a}-m_{13a}\;k_{2}=u_{b}m_{23b}-m_{13b}$$

Equation (9) is the measurement model of the contact wire geometry parameters based on stereovision via triangulation. To perform the dynamic measurement, the pixel coordinates of the contact wire in the two line-scan cameras, *u*_a_ and *u*_b_, are extracted through image processing. Then, the positions of the contact wire in the world coordinate system, [*X*_w_, *Z*_w_], are calculated using Equation (9) and the calibrated parameters of the intrinsic parameter matrix, ***M***_a_ and ***M***_b_, of the two line-scan cameras.

In the overlapping section [1], not only the two contact wires, but also the messenger wires located above the contact wires, would be captured. As mentioned above, when there is one target in the FOV (field of view) of the line-scan camera, the world coordinate of the target could be calculated by using the imaging coordinate of the target in the images of two cameras. However, when the number of the targets is more than one, it is necessary to find the corresponding points of the same target between the stereo image pairs, that is, to solve the stereo-matching problem. Various research studies have been conducted to determine the accurate corresponding points, such as feature descriptors, interest point detectors, and epipolar constraint method [14,22].

This article proposes a matching method based on the position feature of the target. The third line-scan camera is adopted to overcome the correspondence problem. The target imaging coordinate information of the third line-scan camera is used to check the matching of corresponding points in the first two line-scan camera images, which could eliminate the uncertainty caused by the matching of binocular images.

**Figure 4:**
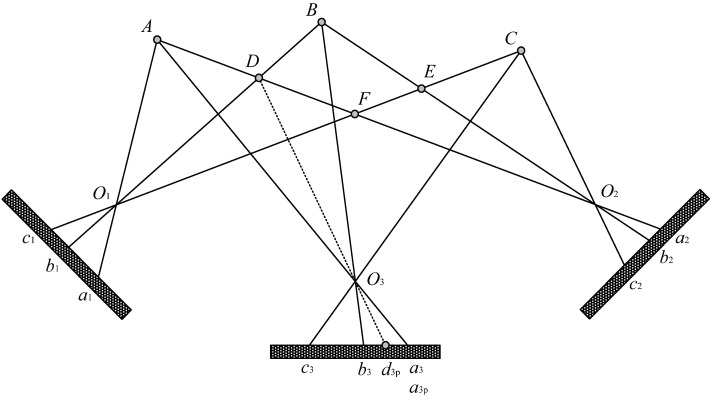
Stereovision matching method using the third line-scan camera.

The proposed matching method is illustrated in Figure 4. *A*, *B*, and *C* are three targets in the FOV of the stereovision system; *O*_1_, *O*_2_, and *O*_3_ are the principal points of the three camera lenses; *a*_1_, *b*_1_, and *c*_1_ are the imaging points of the three targets in the left camera; *a*_2_, *b*_2_, and *c*_2_ are the imaging points of the three targets in the right camera; and *a*_3_, *b*_3_, and *c*_3_ are the imaging points of the three targets in the middle camera. The matching procedure operates in three steps, which are described as follows.

Step 1: All possible spatial points are reconstructed based on the extracted image coordinate data of the left and the right cameras, using the enumeration method. Among the possible spatial points shown in Figure 4, *A*, *B*, and *C* are the real targets, while *D*, *E*, and *F* are the false targets. Then, the key to the matching procedure is to verify all of the reconstructed spatial points.

The verification of points *A* and *D* is illustrated bellow. Point *A* is reconstructed using the imaging point *a*_1_ of the left camera and *a*_2_ of the right camera. Point *D* is reconstructed using *b*_1_ of the left camera and *a*_2_ of the right camera.

Step 2: The reconstructed points *A* and *D* are re-projected onto the imaging chip of the middle line-scan camera and form the re-projected imaging points *a*_3p_ and *d*_3p_. The re-projection imaging coordinates of points *a*_3p_ and *d*_3p_ are denoted as *u*_a3p_ and *u*_d3p_, respectively.

Step 3: The imaging coordinates of *a*_3_, *b*_3_, and *c*_3_ are denoted by *u*_a3_, *u*_b3_, and *u*_c3_. The validity of the re-projected imaging points *a*_3p_ and *d*_3p_ is verified by calculating the distance between the extracted image coordinate data and the re-projected imaging coordinate data. An appropriate threshold, *T*, is adopted by taking into account the average deviation distance of re-projection of the line-scan camera. With regard to the re-projected imaging point *a*_3p_, the distance between *u*_a3_ and *u*_a3p_ is less than the threshold (*T*), verifying the effectiveness of reconstructed point *A*. However, reconstructed point *D* is judged as a false target since there is no candidate imaging point near the re-projected imaging point *d*_3p_.

The verification-procedure speed in this study is fast and satisfies the requirement of real-time measurement. In addition, the matching method is robust for both day and night inspection since the matching precision is not easily affected by the changes in ambient lighting.

### 3.2. Determining the Wear Width of the Contact Wire

**Figure 5:**
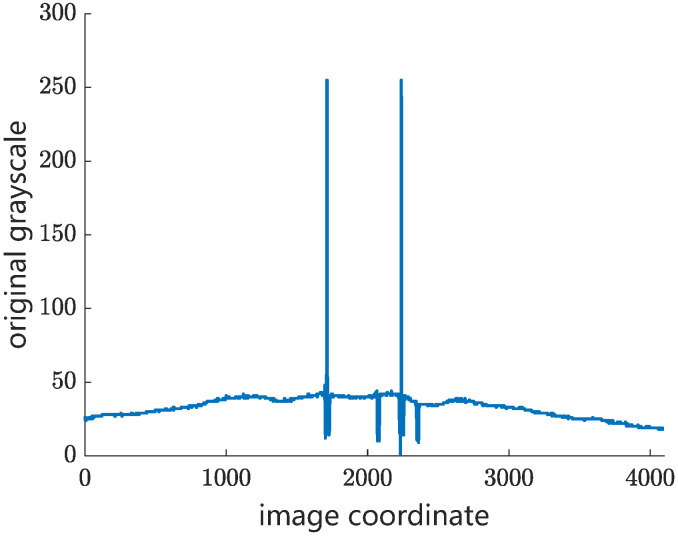
Original grayscale curve of catenary in an overlapping section.

**Figure 6:**
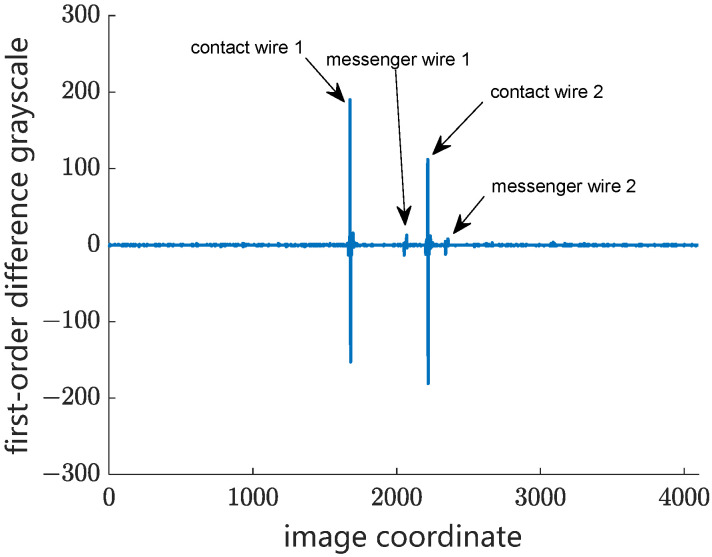
First-order difference of grayscale curve of catenary in an overlapping section.

Determining the wear width of the contact wire includes two steps, the extraction of wear width in pixels from the image and the calculation of the physical wear width in mm. The specific steps of extracting the wear width in pixels from the image are as shown below:

Step 1: The candidate wires are extracted by using the edge detection operator. The first-order difference of grayscale curve is designed to detect the candidate objects in different weather conditions. Figure 5 shows the original grayscale curve of catenary on a cloudy day in an overlapping section. Therefore, there are two peak-shaped regions and two valley-shaped regions in the background grayscale curve, where the peak-shaped regions are the two contact wires in the overlapping section and the valley-shaped regions are the two messenger wires. Figure 6 shows the processed images by using the first-order difference operator. The grayscale gradient of the contact wire is obviously higher than that of the messenger wire, which helps to minimize the interference from the messenger wire in matching.

Step 2: The peak-shaped regions and the valley-shaped regions are extracted from the fitting background grayscale curve. Mean filter operator is adopted to acquire the fitting background grayscale curve and minimize the influence of noise. Then, the boundary between the contact wire and the background is carefully determined. An empirical threshold of the pixel width of a contact wire is used to assist in determining boundaries. The threshold is determined by experimental statistics, and the value of the threshold is affected by the resolution of the line-scan camera, the FOV of the camera and the height variation in the contact wire.

**Figure 7:**
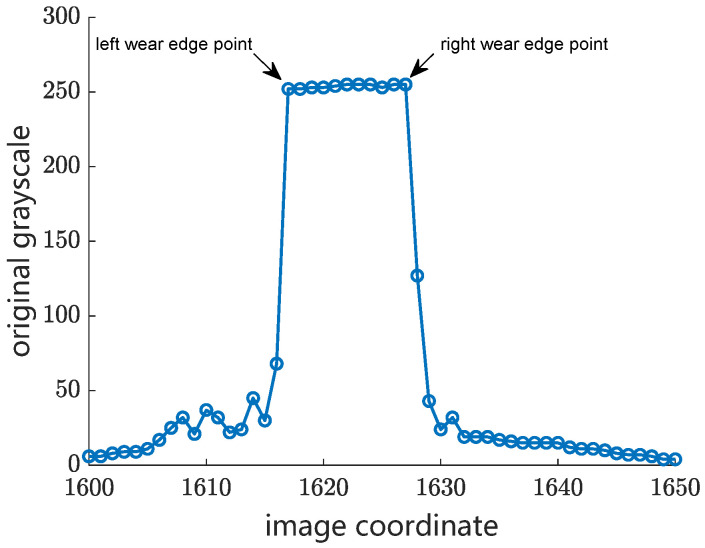
Original grayscale curve of a contact wire with a wear surface.

Step 3: The left and right edge points of the wear surface are located within the peak-shaped region of a contact wire in the image. As mentioned above, the wearing surface has much higher light-reflecting characteristics than the lateral surface of the contact wire. As a result, the grayscale of the wear surface region is several times higher than that of the rest of regions of the contact wire. As shown in Figure 7, the grayscale of the lateral surface regions of the contact wire decreases rapidly from the wear edge point to the background. In this case, the second-order difference of the grayscale curve is adopted to locate the left and right wear edge points within the peak-shaped region of the local grayscale curve of the contact wire. The wear width in pixels is extracted by calculating the difference of the coordinates between the left and the right wear edge points. Meanwhile, the central point of the wear surface is determined.

Step 4: The real targets in the FOV are determined by using the stereovision matching method proposed in this study. Several targets may be extracted in an overlapping section in daytime inspection, i.e., the operating contact wire, the non-operating contact wire, and the two messenger wires which support these two contact wires. The position of each target in the world coordinate system is calculated by triangulation, using Equation (9).

Step 5: The messenger wires are excluded based on the fact that the height of the contact wire is lower than that of the messenger wire. A target-tracking operator is used to exclude the other interference targets, such as the droppers and the cantilevers of the support system. This is due to the fact that the contact wire is continuous along the railway line, while the projections of both the droppers and the cantilevers on the image are discontinuous in the direction of the railway line.

**Figure 8:**
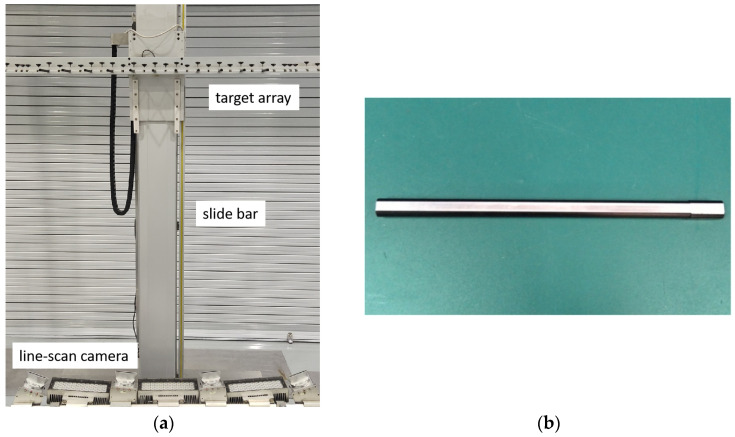
Calibration tool of image resolution: (**a**) calibration tool setup and (**b**) a target with a wear surface.

The calculation of the physical wear width in mm relies on the determination of the image resolution in the measurement range. For different types of electrified railways in China, the height from the car roof to the contact wire would be in the range between 1300 mm and 2500 mm. The stagger would vary from −400 mm to 400 mm in an electrified railway to avoid the continuous friction at the same point of the pantograph. If the effect of the stagger variation is not taken into account, a significant error would be introduced when considering the image resolution. In this study, the image resolution in various sections of measurement range is carefully calibrated using a calibration tool, which takes into account the impact of both the height variation and the stagger variation. As shown in Figure 8a, a set of horizontally arranged targets is fixed on top of a slide bar, with a spacing of 100 mm between each target. A flat surface with a width of 6 mm is fabricated on the bottom of each target, which simulates a contact wire with a wear surface, as shown in Figure 8b. Each target in the horizontal array denotes a contact wire with a different stagger value. The height of the target array could be set in the measurement range by moving the slider bar up and down.

**Figure 9:**
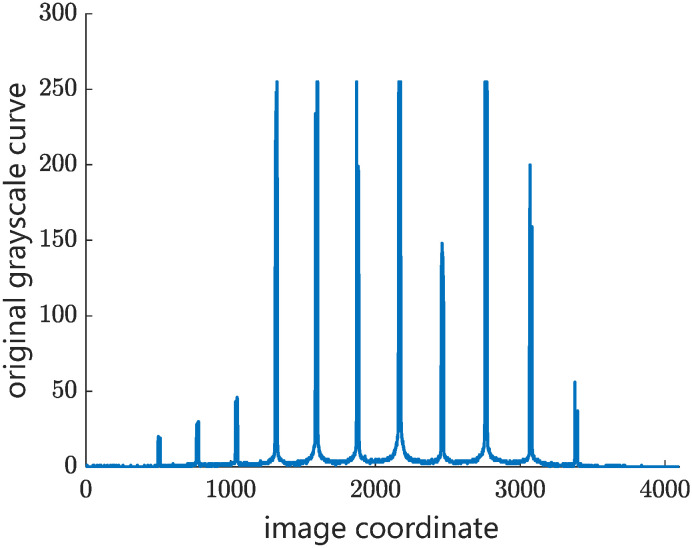
Grayscale curve of the arranged targets of the calibration tool.

First, the parameters of the matrix (***M***) in Equation (8) of each line-scan camera are calculated using multiple sets of calibration data, including the world coordinate data, [*X*_w_, *Z*_w_], and the corresponding imaging coordinate data, *u*. After that, the wear width of each target is extracted in pixels from the captured grayscale curve, using the above image-processing procedure, as shown in Figure 9.

The image resolution with a fixed position is calculated by dividing the preset physical width of 6 mm by the extracted image width of the wear surface, with the unit of mm/pixel. Table 1 illustrates the calibrated image resolutions of the second line-scan camera at different horizontal positions with a height of 1300 mm from the car roof. The image resolutions of each line-scan camera at different horizontal or vertical positions are determined by repeating the above calibration. Thus, the measurement range is divided into an array of small square areas. The image resolution within the same small square area is considered to be uniform. As a result, the distribution of the image resolution in the measurement range is determined by using this lookup-table method. Then, the physical wear width is calculated accordingly. Finally, the residual thickness, *h*, of the contact wire is calculated according to the wire type and the rated cross-section diameter of the contact wire by using Equation (1).

### 3.3. Measurement Apparatus Design

**Figure 10:**
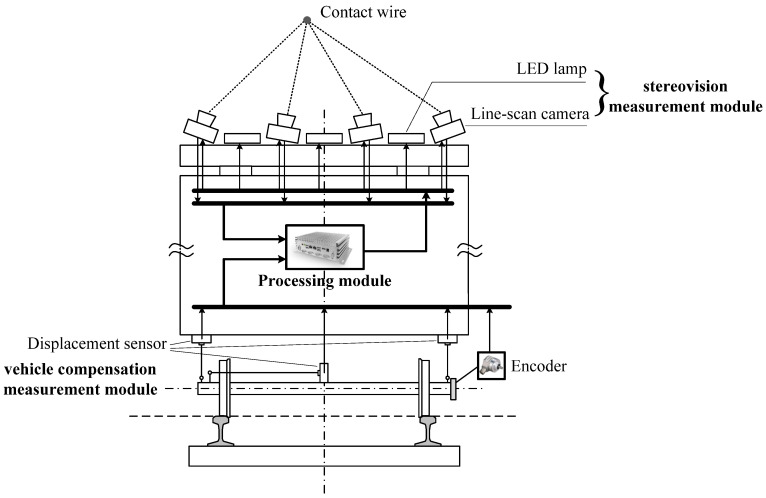
Architecture diagram of the measurement apparatus.

The architecture diagram of the measurement apparatus based on the measurement method for contact wire wear in this study is shown in Figure 10. The measurement apparatus is composed of the stereovision measurement module on the train roof, the processing module inside the train, and the vehicle compensation measurement module under the train. The stereovision measurement module consists of four line-scan cameras with different angles and three LED lamps. The vehicle compensation measurement module includes three displacement sensors. When the train sways, two sensors are used to measure both the left and the right vertical displacement of the train relative to the rail surface, and then the roll angle of the train is calculated by using the displacement data of these two sensors and the width of the train; the third sensor is applied to measure the horizontal displacement of the train relative to the center line of the track. The processing module processes the image data of catenary captured by the line-scan cameras and the vehicle compensation data and calculates the geometry parameters (stagger and contact wire height) and the wear parameters (residual thickness, *h*; or worn area, *A*). The processing module receives the distance pulses produced by the photoelectric encoder, which is installed on the train wheel and triggers all the line-scan cameras to exposure simultaneously.

**Figure 11:**
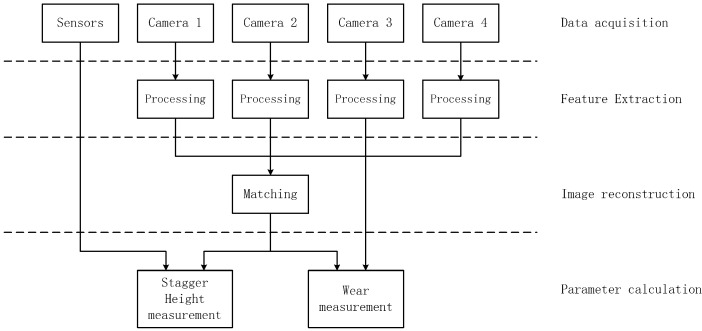
Architecture diagram of the processing module.

The flowchart of the processing module to perform the measurement of the contact wire wear is shown in Figure 11, which includes four steps:

Step 1: Acquire the image data of the four line-scan cameras and the voltage data of the three displacement sensors.

Step 2: Extract the features from each line-scan camera’s image, including the central point coordinate and the wear width of the candidate targets.

Step 3: Execute the stereovision matching algorithm to determine the corresponding points of the contact wire among the stereo images, and then calculate the position of the contact wire in the train roof coordinate system.

Step 4: Calculate the position of the contact wire in the rail surface coordinate system by using the calculated position data from Step 3 and the measurement data of the vehicle compensation measurement module based on the Euclid-space transformation. Then, the physical wear width of the contact wire is determined by using both the data of the wear width in pixels from Step 2 and the image resolution data corresponding to the current position.

The catenary image quality is essential to the wear measurement of the contact wire. In this study, a new kind of high-speed synchronized stroboscopic lighting technology is developed as an alternative active light source. The high-speed synchronization of lighting and camera exposure is realized by a same-trigger pulse. The imaging and lighting devices are able to work stably at 1000 Hz in the pulsed mode, which ensures the dynamic measurement of contact wire wear on the inspection train. Since the duty cycle of the pulsed lighting is low (less than 0.1), the power consumption of the light source is greatly reduced, which is no more than 5% of the spotlights’ total power in our previous research [19,20]. Compared with the continuous LED lighting, the thermal performance of the LED chip in stroboscopic mode is significantly improved, which greatly extends the lifetime of the light source. Moreover, compared with the continuous intensity lighting with rated current, an over-driven pulsed current is used to obtain a light beam with higher luminous flux, which highlights the image features of the wear surface of the contact wire.

**Figure 12:**
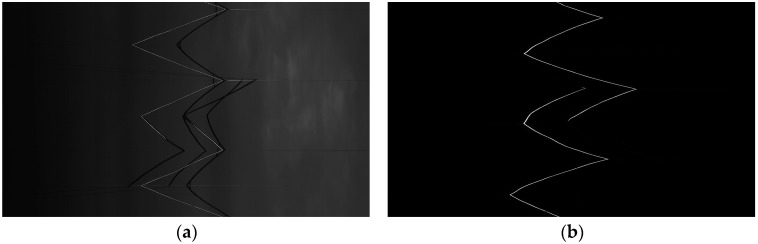
Comparison of catenary images illuminated by two types of light sources in daytime inspection: (**a**) white LED lamps and (**b**) blue stroboscopic LED lamps with band-pass optical filters.

Blue LED lamps combined with band-pass optical filters are adopted to minimize the influence of sunlight. A comparison test in daytime was performed on two light sources, that is, the white LED lamp and the blue stroboscopic LED lamp with the optical filter, as shown in Figure 12. In Figure 12a, the light zigzag lines are the contact wires due to the good light-reflecting characteristics, while the dark zigzag lines are the messenger wires due to the poor reflectivity. In Figure 12b, the grayscale of the sky is greatly reduced, and the image SNR in the daytime environment is significantly improved by using the proposed lighting technology. The messenger wires disappear in Figure 12b because of the little difference in grayscale between the messenger wires and the sky. In this case, the number of the extracted candidate targets is reduced, which accelerates the stereo matching.

## 4. Results and Discussion

### 4.1. Experimental Result

**Figure 13:**
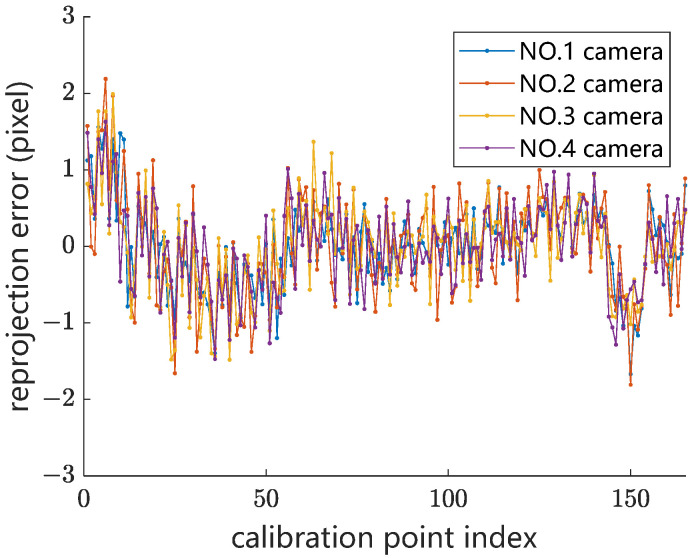
Re-projection error distribution of four line-scan cameras.

The measurement apparatus based on the proposed measurement method was fixed on an inspection train. The four line-scan cameras are the Eliixa+ multi-line high-speed CMOS cameras (Teledyne E2V company, Thousand Oaks, CA, USA), with a resolution of 4096 pixels. Compared with the CCD camera used in the previous research [19,20], the CMOS camera used in this study eliminates the blooming effect and smear effect under strong light illumination. The lens is an Interlock C 35 mm lens (Carl Zeiss AG, Oberkochen, Germany), with robust full-metal construction and low distortion, and then the angle of FOV for each camera is about 60°. The parameters of the intrinsic parameter matrix, ***M***, are calculated using the proposed calibration tool. Figure 13 shows the re-projection error diagram of the four cameras. The average re-projection error for all calibration points of the four line-scan cameras is 0.42, 0.55, 0.55, and 0.52 pixels, respectively. The measurement accuracy of both stagger and height is within ±3 mm in the train roof coordinate system.

Field tests were performed in National Railway Track Test Center of China Academy of Railway Sciences. The following three groups of experiments were carried out to verify the availability of the proposed measurement method for contact wire wear.

**Figure 14:**
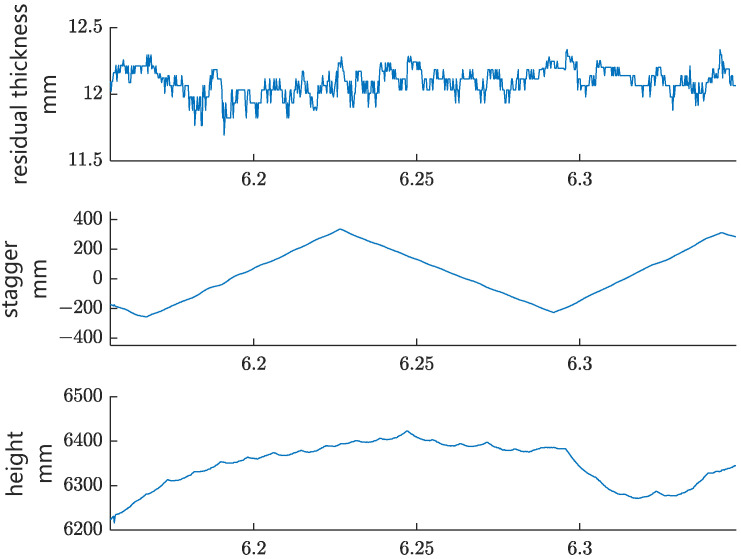
Test results in a straight-line section.

**Figure 15:**
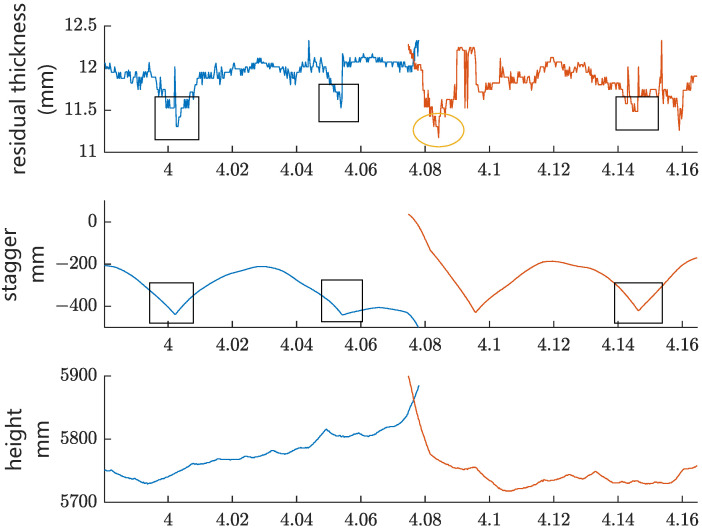
Test results in a curve-line section.

The first group of experiments is functional testing. The type of contact wire in this test is CTA110, and the rated diameter of the contact wire is 12.34 mm. Figure 14 shows the measurement results of residual thickness, stagger, and contact wire height in a straight-line section, while Figure 15 shows the results of these parameters in a curve-line section. The blue and orange lines in Figure 15 represent two contact lines, which belong to two adjacent anchor segments. As shown in Figure 14, the variation in the residual thickness in the straight-line section is relatively small, probably due to the well-distributed elasticity within a span of the straight-line section. In contrast, the minimum residual thickness of each span (except the overlapping section) locates around the catenary mast (refer to the three blue squares in Figure 15) in the curve-line section, which indicates that the distribution of catenary elasticity within a span in the curve-line section is not uniform. This is probably due to the large pull-off force on the contact wire at the mast in the curve-line section with a small curve radius, resulting in better elasticity mid-span than around the mast. The local minimum residual thickness in Figure 15 is located in the overlapping section (refer to the yellow ellipse), probably because of the poor catenaries’ elasticity around the conversion point of the two operating contact wires.

**Figure 16:**
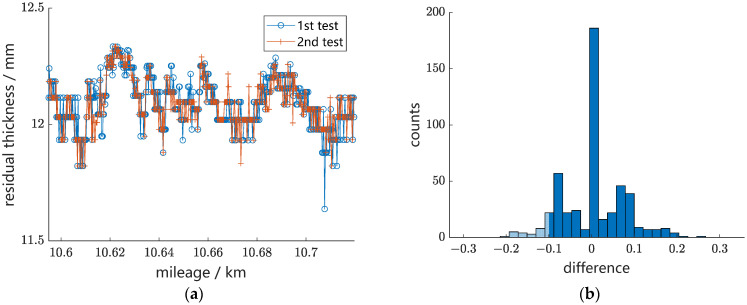
Repeatability verification experiment: (**a**) measurement curves of residual thickness of two tests and (**b**) statistical results of difference between the two tests.

The second group of experiments is the repeatability verification experiment. The round-robin tests of the residual thickness in the same line section present good measurement repetitiveness, which is shown in Figure 16. According to the statistical results of the difference between the two tests shown in Figure 16b, for more than 90% of the measuring points, the absolute difference between the two tests is less than 0.1 mm, indicating that the method presents high repeatability.

The third group of experiments is the accuracy validation test. The type of contact wire in this test is CTA120, and the rated diameter of the contact wire is 12.90 mm. Eight groups of data were collected via a dynamic test and ground measurement, respectively. The difference between the dynamic test data of residual thickness and the ground manual measurement data is listed in Table 2. The average difference value and the maximum difference value are 0.08 mm and 0.13 mm, respectively, which demonstrate that the method has good measurement accuracy. The measurement accuracy of contact wire wear in this study is basically consistent with that reported in the literature [8,9].

### 4.2. Discussion

**Figure 17:**
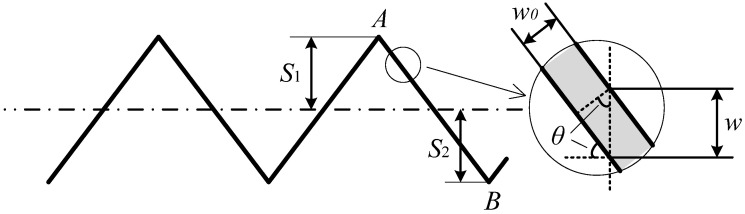
Stagger effect on the measurement of wear width of contact wire in a straight-line section.

Figure 17 shows the stagger effect on the measurement of the wear width of the contact wire. The black solid line with the zigzag shape indicates the stagger distribution along the line. The horizontal dash dot line represents the trajectory of the train roof center during the train’s movement. Then, the measurement plane of the apparatus is perpendicular to the dash dot line. As a result, as shown in the partial enlarged drawing in Figure 17, *w* denotes the measured wear width of the contact wire, *w*_0_ denotes the actual wear width, which is perpendicular to the direction of the contact wire, and there is a small angle *θ* between the measured dot line and the actual dot line.

For the straight-line section shown in Figure 17, points *A* and *B* denote two adjacent masts of the catenary with the design stagger of *S*_1_ and *S*_2_, respectively. The span of the two masts is *L*. Then, the angel, *θ*, can be calculated as follows:

(11) $$\sin\theta =\frac{\left|S_{1}\right|+\left|S_{2}\right|}{L}$$

Then, the relationship between the measured wear width, *w*, and the actual wear width, *w*_0_, is as follow:

(12) $$w_{0}=w\cos\theta$$

For a typical catenary design in a straight-line section in China, *S*_1_ = 300 mm, *S*_2_ = −300 mm, and *L* = 50 m. Then, we can calculate the value of sin⁡θ; sin⁡θ = 0.0120, and cos⁡θ = 0.9999.

As shown in Figure 15, the stagger curve is arc-shaped in a curve-line section, and the angle, *θ*, varies from one mast to the midpoint of two neighboring masts. The maximum value of *θ* appears next to the mast, while the minimum value of *θ* locates at the midpoint of two neighboring masts and is approximately equal to 0. The maximum value of *θ* could be evaluated by calculating the change in stagger value per length along the catenary. For a typical curve line section, the maximum value of sin⁡θ is about 0.0200, and cos⁡θ = 0.9997.

It may seem that the stagger effect on the measurement of the wear width of the contact wire for a typical straight- or curved-line section is small. However, when the train passes through the turnout, the lateral position of the contact wire relative to the roof center changes rapidly, rather than the case of the normal curve-line section. Future work should be done by performing more experiments to further evaluate the stagger effect on the measurement of the wear width in the special section, such as turnout.

## 5. Conclusions

In order to guarantee the operational safety of the catenary infrastructure of railway lines, this paper develops an optical measurement method of contact wire wear using multi-view stereovision technology. The experimental results demonstrate that this method enables the accurate calculation of both the wear width and residual thickness of the contact wire, exhibiting excellent repeatability and precision. This research offers a promising avenue for future condition-based maintenance of contact wire wear, enhancing maintenance efficiency and safeguarding the integrity of catenary infrastructure. However, dynamic measurements on rolling stock present challenges, as the stagger arrangement can influence wear width measurements. Consequently, a further assessment of this stagger effect in regard to specific railway sections is crucial.

## Figures and Tables

**Table 1:** Calibrated image resolution of the second line-scan camera at different horizontal positions with a height of 1300 mm from the car roof.

Horizontal Position (mm)	Image Resolution (mm/pixel)
−400	0.33
−300	0.32
−200	0.32
−100	0.31
0	0.30
100	0.30
200	0.29
300	0.28
400	0.27

**Table 2:** Comparison between dynamic test results and ground measurement results.

Test Data (mm)	Ground Measurement Data (mm)	Difference (mm)
12.72	12.62	0.10
12.80	12.77	0.03
12.83	12.75	0.08
12.80	12.75	0.05
12.80	12.73	0.07
12.77	12.73	0.04
12.83	12.73	0.10
12.83	12.70	0.13

## Data Availability

Data are contained within the article.
